# Comparative Phylogeography in Rainforest Trees from Lower Guinea, Africa

**DOI:** 10.1371/journal.pone.0084307

**Published:** 2014-01-08

**Authors:** Myriam Heuertz, Jérôme Duminil, Gilles Dauby, Vincent Savolainen, Olivier J. Hardy

**Affiliations:** 1 Université Libre de Bruxelles, Faculté des Sciences, Evolutionary Biology and Ecology, Brussels, Belgium; 2 INIA, Forest Research Centre, Forest Ecology and Genetics, Madrid, Spain; 3 Bioversity International, Forest Genetic Resources Programme, Sub-Regional Office for Central Africa, Yaoundé, Cameroon; 4 Imperial College London, South Kensington Campus, London, United Kingdom; 5 Jodrell Laboratory, Royal Botanic Gardens Kew, Richmond, London, United Kingdom; University of California, Berkeley, United States of America

## Abstract

Comparative phylogeography is an effective approach to assess the evolutionary history of biological communities. We used comparative phylogeography in fourteen tree taxa from Lower Guinea (Atlantic Equatorial Africa) to test for congruence with two simple evolutionary scenarios based on physio-climatic features 1) the W-E environmental gradient and 2) the N-S seasonal inversion, which determine climatic and seasonality differences in the region. We sequenced the *trnC-ycf6* plastid DNA region using a dual sampling strategy: fourteen taxa with small sample sizes (dataset 1, mean n = 16/taxon), to assess whether a strong general pattern of allele endemism and genetic differentiation emerged; and four taxonomically well-studied species with larger sample sizes (dataset 2, mean n = 109/species) to detect the presence of particular shared phylogeographic patterns. When grouping the samples into two alternative sets of two populations, W and E, *vs.* N and S, neither dataset exhibited a strong pattern of allelic endemism, suggesting that none of the considered regions consistently harboured older populations. Differentiation in dataset 1 was similarly strong between W and E as between N and S, with 3–5 significant *F*_ST_ tests out of 14 tests in each scenario. Coalescent simulations indicated that, given the power of the data, this result probably reflects idiosyncratic histories of the taxa, or a weak common differentiation pattern (possibly with population substructure) undetectable across taxa in dataset 1. Dataset 2 identified a common genetic break separating the northern and southern populations of *Greenwayodendron suaveolens* subsp. *suaveolens* var. *suaveolens*, *Milicia excelsa*, *Symphonia globulifera* and *Trichoscypha acuminata* in Lower Guinea, in agreement with differentiation across the N–S seasonal inversion. Our work suggests that currently recognized tree taxa or suspected species complexes can contain strongly differentiated genetic lineages, which could lead to misinterpretation of phylogeographic patterns. Therefore the evolutionary processes of such taxa require further study in African tropical rainforests.

## Introduction

The rich biodiversity of tropical rainforests results from the complex interaction of continental drift, past climate oscillations, dispersal events and adaptive processes. The rainforests of Atlantic Equatorial Africa, ranging from Liberia in the West to Gabon in the East, are among the World’s major biodiversity hotspots [1]–[3]. These forests suffer from deforestation and habitat degradation [4], [5], yet the evolutionary processes that shaped their diversity have received relatively little attention [6].

Comparative phylogeography, which examines the distribution of genetic lineages across co-distributed taxa, is an effective approach to assess the evolutionary history of biological communities. Genetic lineages bear the signal of past range dynamics and population size fluctuations (e.g., population bottlenecks or expansion), enabling researchers to test whether genetic data are compatible with defined evolutionary scenarios [7]–[9]. Congruent genetic patterns across taxa of a community are indicative of a similar response to historical events such as climatic oscillations or vicariance events [9], [10]. Such approaches have demonstrated for example a (i) congruent signal of population size decline in Panamanian rainforest trees [11], and (ii) a congruent phylogeographic pattern in South American forest organisms [12], in European Alpine herbs as a function of substrate affinity [13] and in European forest trees, reflecting northward post-glacial recolonization after glacial isolation in southern refugia [10], [14].

In this paper, we focus on the processes that may have shaped the genetic diversity of co-distributed rainforest tree taxa in Africa’s Lower Guinea (LG) phytogeographic region using a comparative phylogeographic approach. LG is the part of the African rainforest that encompasses forests from Southern Nigeria, Cameroon, Equatorial Guinea and Gabon to the south-western part of the Republic of Congo. It is one of three sub-centres of plant endemism – together with the Upper Guinea (UG, Sierra Leone to Ghana) and the Congolia (C) – of the Guineo-Congolian tropical forest phytogeographic region [15], which harbours ca. 6400 endemic species [1], [16]. Two types of physio-climatic features can have affected the distribution of biodiversity at the regional scale in LG, 1) the west-east environmental gradient, and 2) the north-south climate hinge, and both can have influenced the distribution of within-taxa genetic diversity in complex ways through evolutionary time [17]–[19].

The LG rainforests reflect a west-east (W-E) environmental gradient, with generally evergreen forests located close to the coast of the Gulf of Guinea (W) and semi-evergreen forests inland (E) [19]–[21]. The Atlantic forests are strongly affected by the monsoon regime and receive high annual rainfall (above *ca.* 2000 mm/year), lower insolation (ca. 1300 hours/year) and have less marked seasonality than inland forests, which receive *ca.* 1600–2000 mm precipitation per year and up to 2000 hours of sunlight [17]. Atlantic forests show both high species richness and endemism, especially those of southwestern Cameroon [3], [22]–[25]. The W-E environmental gradient may have influenced the distribution of biodiversity in conjunction with the Plio-Pleistocene climate oscillations. Fossil pollen evidence from marine and lake cores indicates that African tropical forests have been repeatedly fragmented while savannah vegetation expanded during the Plio-Pleistocene cold and dry stages [26]–[29]. Palaeoecological and species distribution data led to the suggestion that many rainforest taxa survived the cold and dry stages essentially in the comparatively moister, mountainous Atlantic region of LG [18], [23], [30]–[33]. These W-E regional settings lead to two contrasting expectations for W-E genetic diversity patterns within taxa in LG: If separate populations occurred in the W *vs.* E regions, for instance in the case of old differential adaptation to water availability and/or past history, we would expect differentiation between Atlantic (W) and inland (E) forests. Allelic endemism patterns could then emerge in either the W or E depending on the age of the differentiated populations. For instance, the distribution of distinct sister species of *Erythrophleum* (Fabaceae) close to the Bight of Bonny (W) and inland (E) concurs with this expectation [34]. Alternatively, current W and E populations could be derived more recently from the same regional population, for instance if populations persisted in the W and population extinction occurred in the E in recent glacial cycles [18], [33]. In this case we would expect only weak differentiation between regions and a pattern of higher allele endemism in the ancient vs. the more recently colonized range part of the rainforest taxa, because only a subsample of the original allelic diversity is expected to colonize (e.g., [35]).

The second feature that characterizes the LG region is the climate hinge, or latitude of N-S seasonal inversion, which runs roughly parallel to the equator between ca. 0° and 3° N. During the boreal summer (June-August), the northern part of the LG forest (i.e., Cameroon) receives increased rainfall while in the southern part (Gabon) an overcast sky limits evapotranspiration in the absence of rain. In the austral summer, southern Gabon receives heavy rainfall while dry continental winds produce a dry season in the northern fringes of the forest block [17]. Most of the tree taxa included in our study bear fruit in the boreal summer in Cameroon, whereas the phenology is shifted on average by six months in the Gabonese populations. This shift in phenology may have limited gene flow between both regions, and the N-S differences in seasonality may be at the origin of adaptive differences that hinder establishment in the non-native zone (e.g., [36], [37]). These N-S regional settings would lead to the expectation of a pattern of genetic differentiation between populations located respectively N and S of the climatic hinge. The Plio-Pleistocene rainforest history (see previous paragraph) may have enhanced the expected N-S pattern if distinct glacial populations existed N and S of the climatic hinge (e.g., following [18], but not [33]). For instance, *Greenwayodendron suaveolens* (Annonaceae) displays N-S differentiation in LG [38].

In this study we examined two alternative evolutionary scenarios by characterizing the genetic diversity and its spatial structure at maternally-inherited plastid DNA markers in fourteen tree taxa in the LG. We asked the question whether one of the before-mentioned physio-climatic features, the W-E environmental gradient, or the N-S seasonal inversion, was strong enough to have imprinted a very general geographic pattern of genetic variation at maternally-inherited genomes in Central African trees. To capture genetic signals that may have resulted from Plio-Pleistocene or still on-going events, we focused on low-level taxa, usually taxonomic species. Early phylogeographical studies identified considerable levels of lineage divergence within taxonomic species in central Africa and other tropical regions [12], [38], [39], suggesting high levels of undocumented species diversity. Given the paucity of well-established knowledge on the evolution and species delimitation of African rainforest trees, we opted for two complementary sampling strategies. (i) First, we considered fourteen tree taxa with limited sample sizes (12 to 20 individuals) and possibly encompassing a wide range of phylogenetic depths (dataset 1); i.e. some of these taxa may not match the biological species concept, containing more than one genetic species according to current taxon delimitation. This sampling allowed us to assess whether a very general phylogeographic pattern emerges given a wide range of phylogenetic depths among different lineages. (ii) Second, we analyzed larger sample sizes (66 to 169 individuals) in four tree species that are taxonomically very well studied (dataset 2). This sampling provided increased power to detect particular shared phylogeographic patterns among the four species, though at the cost of generality. While several phylogeographic studies have examined the distribution of genetic diversity in tree species from LG [34], [38], [40]–[45], no study to our knowledge has compared diversity using the same plastid marker across taxa or attempted a multi-taxa analysis.

## Materials and Methods

### Ethics Statement

We sampled fourteen common rainforest tree taxa typically occurring in evergreen and semi-evergreen rainforests of the Lower Guinea. Sampling sites included national parks and public land. Sampling permits were obtained from the CENAREST (Gabon) and the Ministry of Scientific Research and Innovation (Cameroon) and sample collection was facilitated by the Wildlife Conservation Society (La Lopé, Gabon), the CIRMF (Franceville, Gabon), the logging company Pallisco (Cameroon), the Missouri Botanical Garden and the NGO Nature Plus. According to IUCN, *Baillonella toxisperma* Pierre (Sapotaceae) is listed as “vulnerable A1cd ver 2.3″, *Milicia excelsa* (Welw.) C.C. Berg (Moraceae) as “lower risk/near threatened ver 2.3″, and *Greenwayodendron suaveolens* (Engl. & Diels) Verdc. as “least concern”, while there is no data on the remaining taxa.

### Sampling Strategy and Molecular Methods

**Figure 1:**
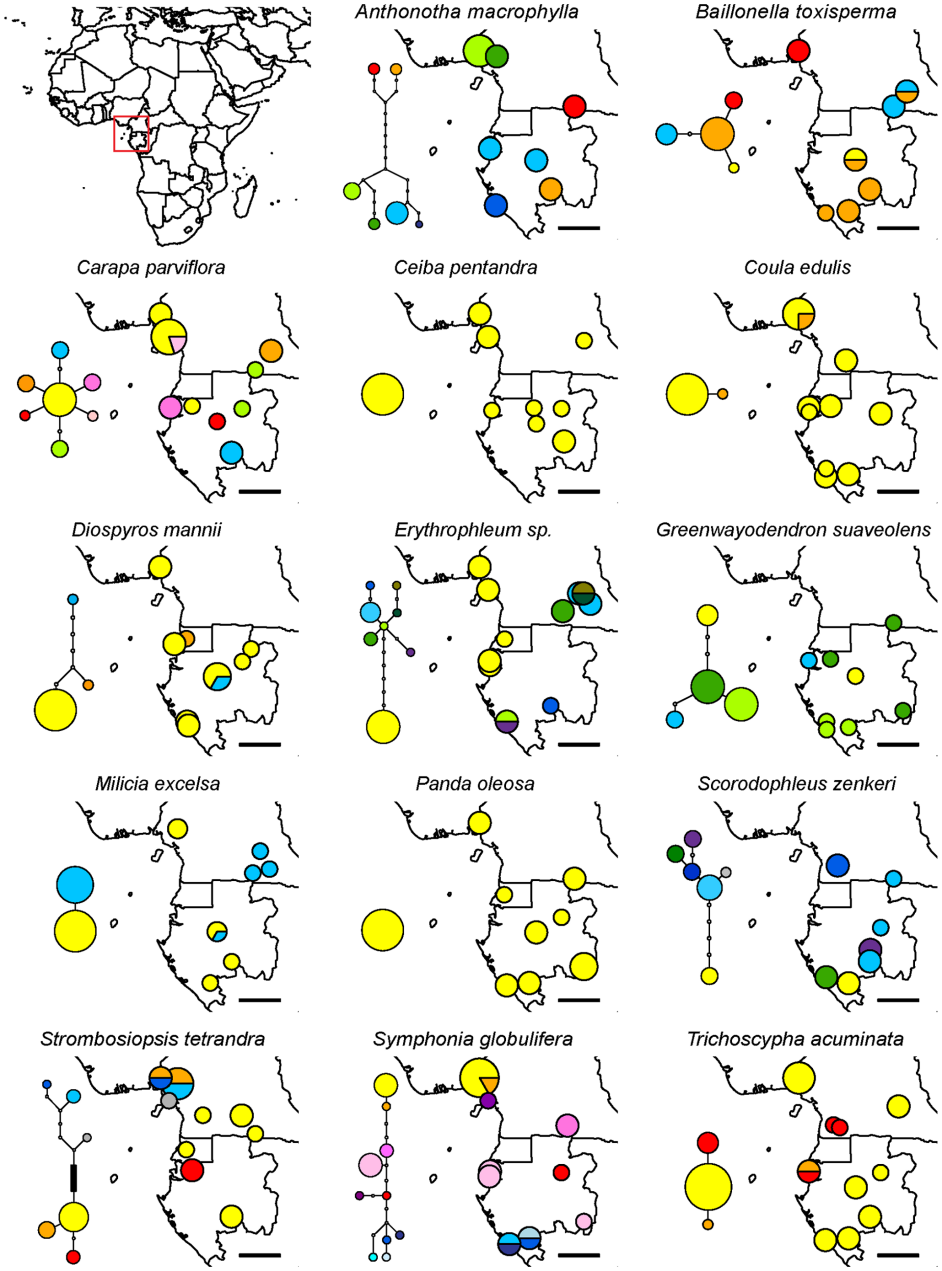
Haplotype maps of 14 Afrotropical tree taxa (dataset 1). Sizes of circles are proportional to the sample sizes of each population, haplotypes are colour-coded. Statistical parsimony networks of haplotypes are included for each taxon: each link represents a single mutation, white circles indicate unobserved putative haplotypes and the black box on the *Strombosiopsis tetrandra* network represents 26 mutations. The scale bar on the maps corresponds to 200 km.

**Figure 2:**
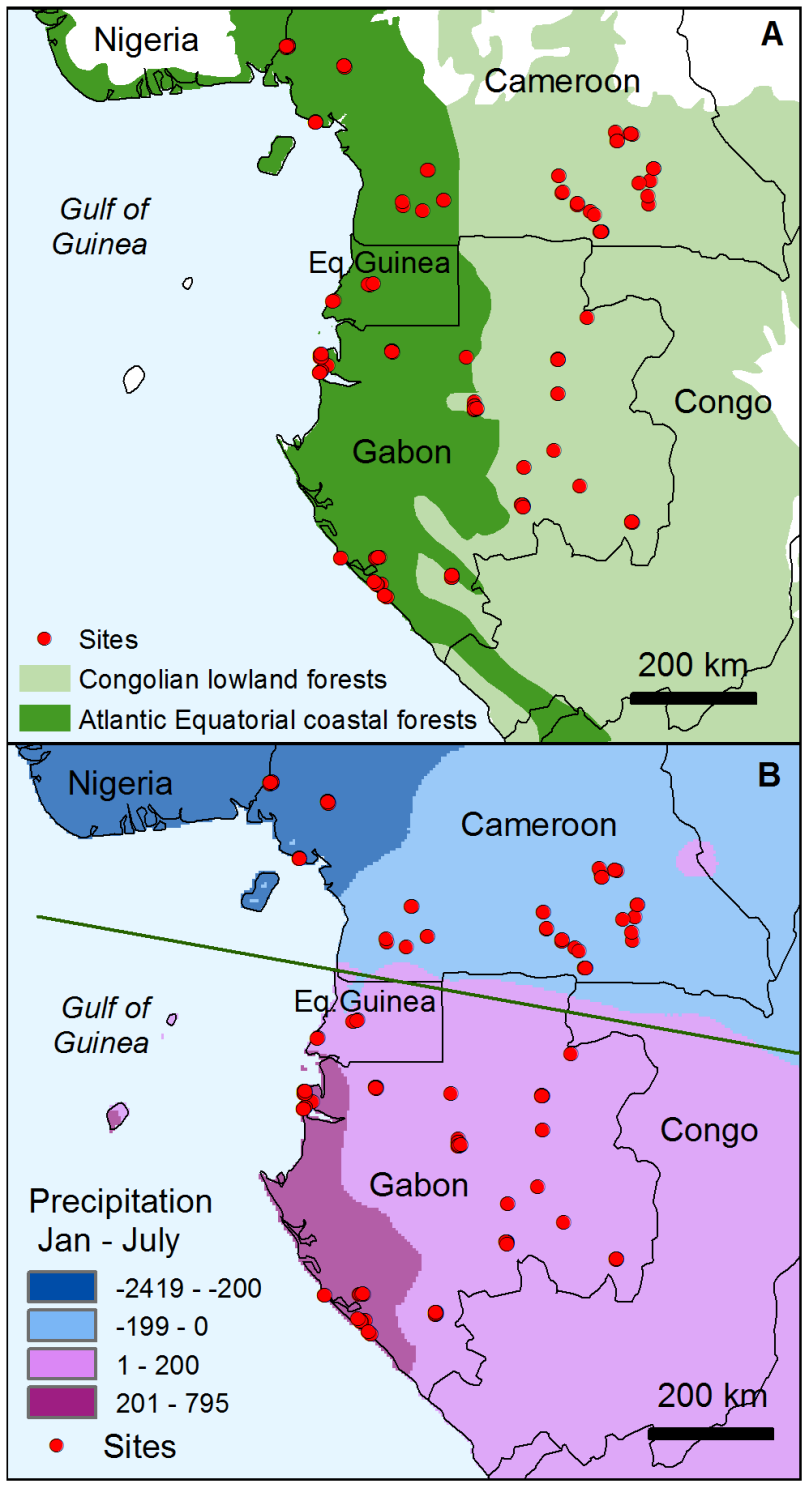
Two physioclimatic features of the Lower Guinea region that led to the formulation of alternative evolutionary scenarios for Afrotropical tree taxa. A) The W-E environmental gradient, B) the N-S seasonal inversion, illustrated by the difference of January and July precipitation (mm).

In a first stage (dataset 1), we sampled fourteen common tree taxa typically occurring in evergreen and semi-evergreen rainforests of the LG (Table 1, Figure 1, Table S1). Thirteen of the sampled taxa are considered as species according to most taxonomic reference books (e.g., Flore d’Afrique Centrale, Flore du Gabon, Flora Zambesiaca) whereas *Erythrophleum* harbours two vegetatively similar and closely related sister species traded under the same timber name (tali) [34]. However, recent or on-going taxonomic revisions or genetic work suggest that the LG study region harbours several species in the genera *Carapa* [46], [47], *Strombosiopsis* ([48], F.J. Breteler, pers. com.) and *Greenwayodendron* [38]. Current knowledge is insufficient to establish whether any of the used taxa matches the biological species concept, and there is evidence that several taxa correspond to closely related species or species complexes (e.g., *Erythrophleum* [34], *Carapa* [47]). Hence, the patterns of genetic diversity observed should include a relatively wide phylogenetic depth, from within-species (in the biological sense) to within complexes of closely related species. Studying plastid DNA patterns of diversity and structure in species complexes should generally be a valid approach because the genealogy of a non-recombinant DNA fragment is not directly dependent on speciation processes [47]. By using a comparative phylogeographic approach across 14 taxa, we were interested in identifying a general pattern across taxa, notwithstanding one or the other outlier potentially caused by geographically delimited evolutionary processes or sampling bias associated to small sample sizes in species complexes. For each taxon, leaf or cambium samples were collected at 7–11 geographic locations (Table 1, Figure 1, Table S1), in a way to include coastal and inland populations, as well as populations north and south of the climate hinge (Figure 2; see also [36]). Taxa represent a wide range of life history traits with respect to successional behaviour, seed dispersal syndromes, size of geographic range and tree strata in the rainforest communities ([49]–[54], Table S2).

**Table 1:** Diversity patterns at the *trnC-ycf6* region in 14 Afrotropical tree taxa.

										Point mutations only				All polymorphic characters			
Taxon	Family	N	pops	L_tot_	L	S(singl.)	inv.	indel	SSR	haps	Hd	π_T_	*θ*_W_	haps		h	v
*Dataset 1*																	
*Anthonotha macrophylla* P Beauv.	Fabaceae(Caesalpinioideae)	17	8	1022	787	12(0)	0	10	1	6	0.824	5.14	4.51	6	0.905		0.394
*Baillonella toxisperma* Pierre	Sapotaceae	13	7	759	721	1(0)	1	0	2	2	0.385	1.07	0.89	4	0.750		0.342
*Carapa parviflora* Harms	Meliaceae	17	9	741	579	4(1)	0	4	0	5	0.647	1.35	2.04	7	0.920		0.210
*Ceiba pentandra* (L.) Gaertn.	Malvaceae	11	8	600	576	0(0)	0	0	0	1	0.000	0.00	0.00	1	0.000		0.000
*Coula edulis* Baill.	Olacaceae	18	9	853	598	0(0)	0	1	0	1	0.000	0.00	0.00	2	0.071		0.071
*Diospyros mannii* Hiern	Ebenaceae	14	8	794	621	5(3)	0	0	2	3	0.275	1.54	2.53	3	0.133		0.114
*Erythrophleum ivorense* A. Chev./*E*. *suaveolens* (Guill. & Perr.) Brenan	Fabaceae(Caesalpinioideae)	20	11	1002	917	9(2)	0	2	2	6	0.747	4.02	2.74	8	0.806		0.393
*Greenwayodendron suaveolens*(Engl. & Diels) Verdc.	Annonaceae	16	8	1019	977	4(0)	1	0	0	4	0.733	1.47	0.15	4	0.786		0.298
*Milicia excelsa* (Welw.) C.C. Berg	Moraceae	16	7	1020	939	1(0)	0	0	0	2	0.525	0.56	0.32	2	0.571		nc
*Panda oleosa* Pierre	Pandaceae	12	8	915	893	0(0)	0	0	0	1	0.000	0.00	0.00	1	0.000		0.000
*Scorodophloeus zenkeri* Harms	Fabaceae(Caesalpinioideae)	13	8	985	894	7(0)	0	2	1	5	0.821	2.50	2.52	6	1.000		0.380
*Strombosiopsis tetrandra* Engl.	Olacaceae	16	9	887	719	26(0)	0	7	2	6	0.783	13.85	10.90	6	0.875		0.337
*Symphonia globulifera* L.f.	Clusiaceae	19	9	868	688	11(4)	0	1	2	8	0.842	4.83	4.57	10	0.933		0.321
*Trichoscypha acuminata* Engl.	Anacardiaceae	19	9	712	612	1(0)	0	1	0	2	0.281	0.46	0.47	3	0.446		0.232
Overall		15.8	8.4	870	752	5.8 (0.7)	0.1	2	0.9	3.7	0.49	2.63	2.26	4.5	0.585		0.238
*Dataset 2*																	
*Greenwayodendron suaveolens* subsp.*suaveolens* var. *suaveolens* (Engl.& Diels) Verdc	Annonaceae	169	41	963	954	5(1)	2	1	0	5	0.187	0.22	0.75	10	0.668		0.078
*Milicia excelsa* (Welw.) C.C. Berg)	Moraceae	127	20	939	939	2(1)	0	0	0	3	0.399	0.44	0.39	3	0.528		0.280
*Symphonia globulifera* L. f.	Clusiaceae	66	20	912	693	22(9)	0	4	1	22	0.906	5.54	6.67	22	0.974		0.152
*Trichoscypha acuminate* Engl.	Anacardiaceae	73	28	752	547	3(0)	0	1	0	4	0.477	0.96	1.13	5	0.516		0.145

*N*, sample size; *pops*, number of sampling locations; *L_tot_*, total length of alignment; *L*, length of alignment excluding gaps and missing data; *S(singl.)*, total number of single nucleotide mutations (number of singletons); *inv.*, number of inversions; *indel*, number of insertions/deletions; *SSR*, number of simple sequence repeats; *haps*, number of haplotypes; *Hd*, haplotype diversity; *π_T_* and *θ_W_*, estimates of nucleotide diversity x 10^3^; *h* and *v*, genetic diversity based on ordered or unordered alleles; nc, not calculated. For details see materials and methods.

**Figure 3:**
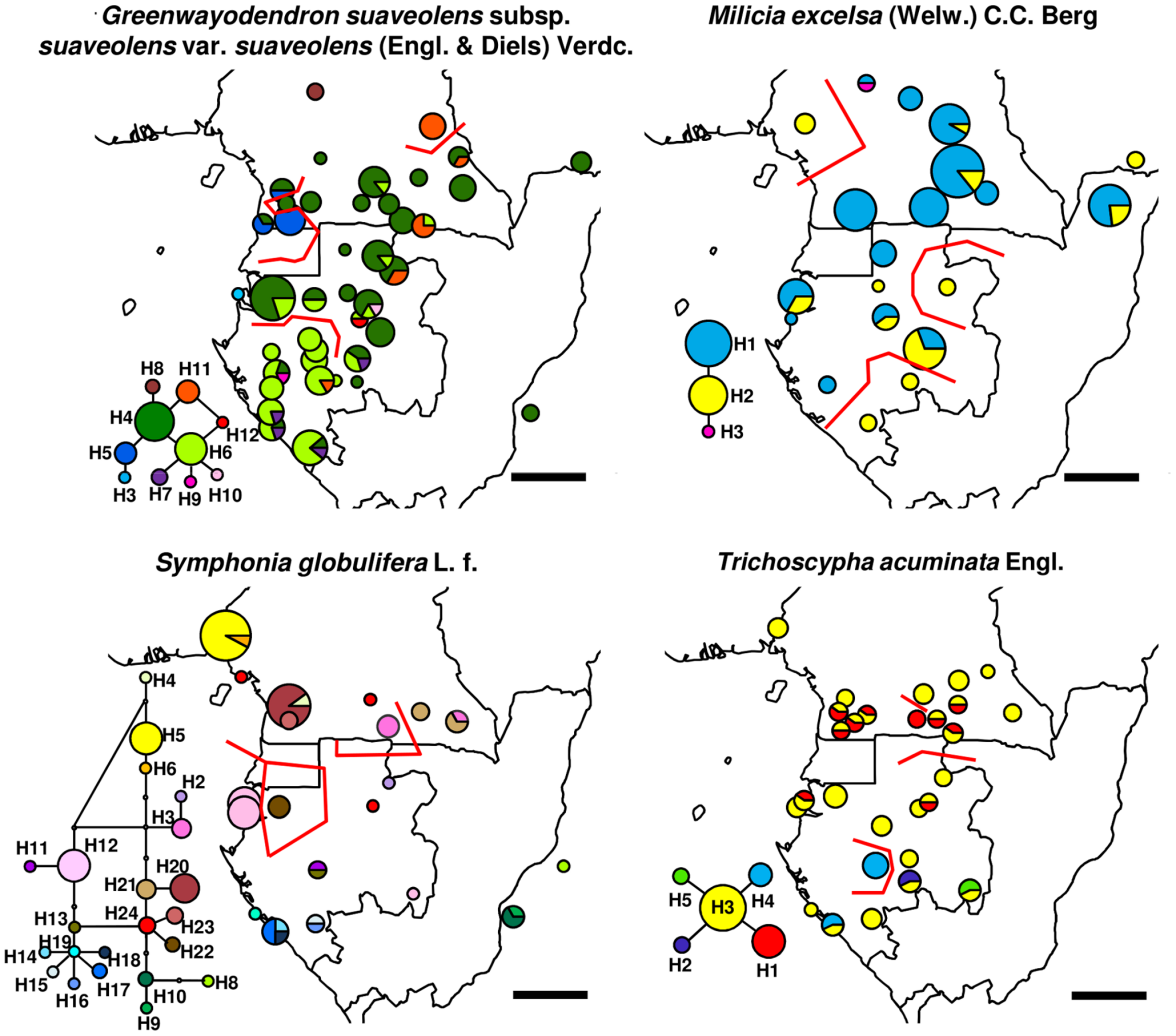
Haplotype maps of four Afrotropical tree species (dataset 2). Sizes of circles are proportional to the sample sizes of each population, haplotypes are colour-coded. Statistical parsimony networks of haplotypes are included for each species: each link represents a single mutation; white circles indicate unobserved putative haplotypes. Red lines correspond to the three strongest genetic barriers (BARRIER program) identified in each species.

In a second stage, we included additional samples for four species (dataset 2, Figure 3): *Greenwayodendron suaveolens* subsp. *suaveolens* var. *suaveolens* (Engl. & Diels) Verdc., totalling n = 169 individuals from 41 locations published by Dauby et al. [38]; *Milicia excelsa* (Welw.) C.C. Berg, n = 127, 19 locations, published by Daïnou et al. [40]; and new data in *Symphonia globulifera* Lf., n = 67, 20 locations and *Trichoscypha acuminata* Engl., n = 73, 28 locations. Complementary information indicated that these taxa most likely matched the biological species concept; specifically, in *G. suaveolens* subsp. *suaveolens*, we only included samples morphologically and genetically identified as var. *suaveolens* which likely represents a distinct biological species from *G. suaveolens* subsp. *suaveolens* var. *gabonica* [38]; in *M. excelsa*, polymorphism was low and no deep lineage divergence was previously identified in LG [40]; in *S. globulifera*, the same samples included here did not display deep lineage divergence when sequenced at another marker (*psba-trnH* region of plastid DNA, [55]) and in *T. acuminata*, although closely related species are present in LG [56], we only included samples which were identified as *T. acuminata* in the field and that displayed closely related haplotypes. Herbarium vouchers were collected generally in duplicate for at least one individual per location when possible and deposited at the National Herbarium of Cameroon (YA) or the Herbier National du Gabon (LBV) and at the herbarium of the Université Libre de Bruxelles (BRLU, see Table S1).

Total DNA was extracted from silica-dried plant material using either a CTAB procedure [57] or the Invisorb® DNA Plant HTS 96 Kit (Invitek, Berlin, Germany). After an initial screening for sequence polymorphism of six plastid DNA (cpDNA) regions [58], we retained the *trnC-ycf6* region because it could be amplified in all taxa and displayed fairly high levels of polymorphism. The *trnC-ycf6* region was amplified in a reaction volume of 25 µL containing c. 20 ng of DNA and 0.625 U Taq polymerase in 1× reaction buffer (Qiagen, Venlo, Netherlands) supplemented with 0.1 µM of each primer (*trnC*: [59], *petN1r*: [60]), 1 mM MgCl_2_ and 0.2 mM of each dNTP. The cycling profile was 94°C for 3 min; 35 cycles of 94°C for 30 s, 50°C for 30 s and 72°C for 1 min 20 s; final extension at 72°C for 7 min and cooling to 10°C. PCR products were purified on filter columns (MSB HTS PCRapace/C(96) kit, Invitek, Berlin, Germany) and sequenced using Big Dye v.3.1 chemistry (Applied Biosystems, Lennik, Belgium) and an ABI3100 sequencer (Applied Biosystems).

### Data Analysis

#### Genetic diversity and relatedness of haplotypes

Sequence trace files corresponding to forward and reverse reads were aligned with CodonCode Aligner 3.0.1. (CodonCode Corporation Dedham, MA, USA). Within-taxon alignments of individual sequences were done with MUSCLE 3.6 [61]. To characterize genetic variation in each taxon, we recorded the number of segregating sites *S*, the number of singletons, the number of insertion/deletion (indel) polymorphisms, inversions and microsatellite (SSR) length polymorphisms. Considering point mutations only, we used DnaSp v. 5.10.1 software [62] to compute haplotype diversity *Hd* and two estimators of the population mutation parameter, *θ* = 4*N*_e_ *µ*, namely, nucleotide diversity (*π*), which represents the average number of nucleotide differences per site between two sequences [63], and Watterson’s estimator *θ*_W_ [64] based on *S*. We then considered all polymorphism types (except in the large *S. globulifera* data set, where only SNPs were considered because complex indel polymorphisms prevented unambiguous coding of all polymorphisms) to define haplotypes and computed a haplotype distance matrix for each taxon, defining the distance between two haplotypes as the proportion of polymorphisms differing between them. Statistical parsimony networks among haplotypes were constructed based on these distance matrices using the program TCS 1.21 [65]. The totality of polymorphisms was used for all further analyses except in the large *S. globulifera* data set. We computed genetic diversity *h* and *v* [66], based respectively on ordered or on un-ordered haplotypes, in SPAGeDi v. 1.3. [67]. The frequencies of haplotypes per sampling location were represented in haplotype maps using ArcGIS 10 software (ESRI, Redlands, CA, USA).

#### Demographic inference, isolation by distance and phylogeographic structure

To test for demographic or spatial genetic signals of population expansion, such as expected if population recovery and/or recolonization after Plio-Pleistocene fragmentation left a genetic footprint, we used the mismatch distribution with 1000 parametrical bootstraps in Arlequin 3.5.1.3 [68], where a significant test means that the null model of population expansion can be rejected. We further investigated phylogeographic structure in each taxon using isolation by distance (IBD) approaches: We first tested for IBD among unordered haplotypes by regressing the kinship coefficient *F_ij_* between pairs of individuals on the logarithm of geographic distance and compared the slope *blog*(*F_ij_*) to its null expectation from random permutations of the sampling locations using SPAGeDi (one-sided test with alternative hypothesis *blog*(*F_ij_*)<*blog*(*F_ij_* permuted)). We then tested whether nearby individuals were more likely to bear closely related haplotypes than individuals chosen at random, performing the same test using the coefficient *Nij* based on ordered haplotypes (O.J. Hardy, unpublished). Finally, we tested for phylogeographic structure by comparing *blog*(*N_ij_*) to its expectation from permuting rows and columns of the genetic distance matrix.

#### Patterns of endemism and differentiation

To test for patterns of allelic endemism and genetic differentiation as expected under two contrasting evolutionary scenarios (see Introduction), we grouped samples into pairs of categories (hereafter regions) and examined levels of genetic diversity and endemism within regions and differentiation between them. For the W-E scenario (1), which emphasizes the response to environmental and climatic conditions (e.g., past population differentiation, or recent common origin in a single region), samples were classified as belonging to generally evergreen Atlantic forests or to semi-evergreen inland forests [19]. For the N-S scenario (2), which emphasizes the role of the climate inversion and seasonality differences across the climate hinge (possibly in conjunction with Plio-Pleistocene forest history, see Introduction), samples were classified as located N or S of the seasonal inversion line (Figure 2). Within each of the regions defined according to the two scenarios, we recorded the number of haplotypes observed (*Nhaps*), the rarefied haplotype richness (*Ar_x_*, the number of haplotypes expected in a sample of size *x*), the number of private haplotypes (*Npriv*) and the rarefied private haplotype richness (*Arpriv*_x_, the number of private haplotypes expected in a sample of size *x*) using the program HP-Rare [69], and we computed genetic diversity and its standard error using the program CONTRIB [70]. In dataset 1, haplotype richness statistics were collected separately for each taxon and for haplotypes pooled across the 14 taxa. In dataset 1, we carried out one-way analysis of variance in the *R* software [71] to test for the effects of taxa and of geographical regions on genetic diversity statistics in each of the two scenarios. We then computed the differentiation statistics *F*_ST_ for unordered and *N*_ST_ for ordered alleles [66] for each scenario (both datasets) and tested against the null hypothesis of no population differentiation using permutation tests in SPAGeDi. The number of significant differentiation tests between regions was recorded for each scenario and the contribution of mutations to differentiation was assessed with permutation tests (*N*_ST_>*N*_ST(permuted)_, [72]). We also evaluated the relative support of both scenarios counting the taxa with stronger differentiation (*F*_ST_) in the candidate vs. alternative scenario (in dataset 1).

**Figure 4:**
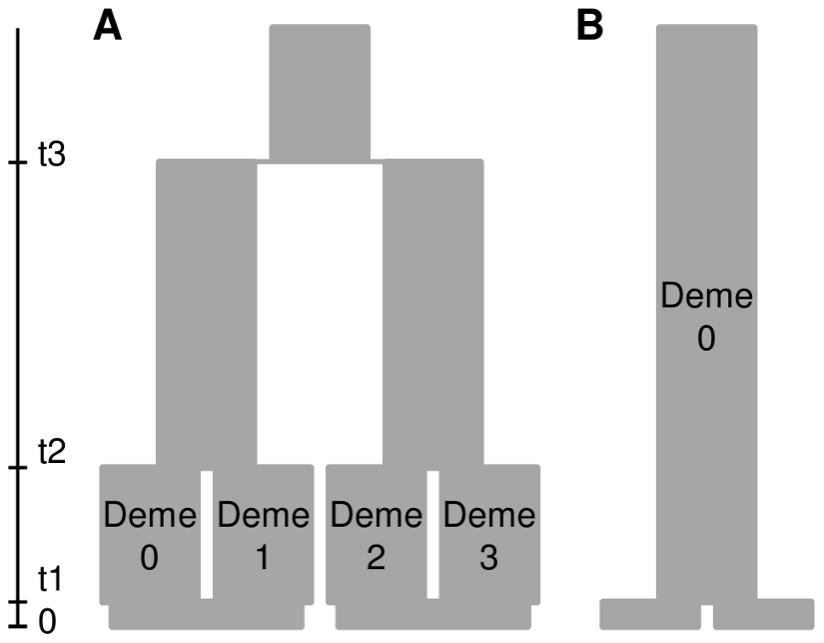
Graphical representation of two evolutionary scenarios tested using coalescent simulations. A) population divergence scenario, B) constant population size scenario.

To assess the power of our approach to identify population subdivision, discriminate between the tested scenarios, and to assess the rate of false detection of population subdivision when it is absent (type I error), we applied a coalescent simulation approach using simulated sample sizes mimicking our initial (n = 8/population) and enhanced data sets (n = 30/population). We used the software fastsimcoal [73] to simulate two simple evolutionary scenarios (Figure 4): in each scenario, two samples of n = 8 or n = 30 genes were taken at generation 0 (present time) in each of two populations, their genealogy was simulated backward in time, and then mutations were added onto the genealogy forward in time. In the “divergence” scenario (Figure 4, A), we modelled one ancient divergence event at t3 = 1600 generations ago, and a more recent divergence at t2 = 200 generations leading to two demes in each population. Deme size was of constant size *N* throughout the genealogy. This model was motivated by the observation of substructure in some species of dataset 1 and could be interpreted as repeated divergence events triggered by recurrent climate oscillations (see [55]), or by other vicariance or local adaptation events. In the “constant” population size scenario without divergence (Figure 4, B), both samples derived from the same deme t1 = 1 generation ago. In each scenario, we simulated a DNA sequence of length 1000 bp and varied the deme size *N* and mutation rate *µ* among runs to obtain average diversity and differentiation levels similar to those observed in our observed dataset 1. We repeated simulations 100 times in each run, and used the software Arlequin 3.5.1.3 in batch mode [68] to compute summary statistics for the simulated data sets. To assess the power of our data to detect genetic structure and to evaluate type I error, we recorded the number of significant differentiation tests (*F*_ST_) respectively in the divergence and constant scenarios and compared them to the observed data. For the differentiation scenario that most closely matched the observed dataset 1, we repeated the simulation recovering n = 4 samples for each of four demes at t0. We then used AMOVA in Arlequin to compute the differentiation between populations with simulated substructure, *F*_CT[(01)(23)]_ (where subscript numbers stand for the four demes), and the differentiation between groups composed of samples from different populations, *F*_CT[(02)(13)]_. This mimicked grouping the samples into W-E *vs.* N-S, with one of the two groupings corresponding to the true divergence history. The fraction of simulations where *F*_CT[(01)(23)]_>*F*_CT[(02)(13)]_ represents the power to identify the correct differentiation pattern between the two groupings considered.

#### Shared spatial patterns of genetic structure

To geographically locate the most important genetic breaks and investigate whether taxa shared patterns of geographic differentiation, we searched for genetic barriers using the Monmonier maximum difference algorithm in the software BARRIER v.2.2 [74]. This analysis was restricted to dataset 2 because small sample sizes would imply low confidence in barrier location in dataset 1. The matrix of pair-wise differentiation (*G*_ST_) between sampling locations was used together with the sampling coordinates to identify the three most important genetic barriers in each taxon. The co-location of barriers was then evaluated across taxa using the line density tool in ArcGis 10 software (see also [75]).

## Results

### Genetic Diversity and Relatedness of Haplotypes

Sequences of the *trnC-ycf6* region were deposited at Genbank under accession numbers KF487767-KF488074 (Table S1). The *trnC-ycf6* region had an average length of 870 bp (600–1022 bp) in dataset 1 and the number of SNPs varied between 0 and 26 across taxa, resulting in π_T_ values ranging from 0 to 0.0139 (mean 0.0026, Table 1). In dataset 1, we detected between one and eight haplotypes per taxon when considering SNPs and between one and ten haplotypes when considering all polymorphic characters, for an average sample size of 16 individuals and eight sampling locations per taxon. Some high-diversity taxa displayed divergent lineages, with some haplotypes differing by six or more mutations without putative intermediate haplotypes: *Anthonotha macrophylla*, *Strombosiopsis tetrandra* and *Erythrophleum ivorense/suaveolens* (Figure 1). The geographic representation of haplotype frequencies did not reveal any obvious common distribution pattern of haplotypes across taxa (Figure 1). In dataset 2, we detected between three and 22 haplotypes per species, for sample sizes of 66–169 individuals and 20–41 locations (Table 1, Figure 3).

### Demographic Inference, Isolation by Distance and Phylogeographic Structure

The null model of demographic expansion was rejected in five, and the spatial expansion model was rejected in two out of 12 taxa with *P*<0.05 in dataset 1 (Table 2). In dataset 2, six out of eight tests rejected expansion models (Table 2). Isolation by distance of haplotypes was found in ten out of fourteen taxa in dataset 1 and in all four species of dataset 2, evidenced by a significant negative regression slope of pairwise kinship, *blog*(*F_ij_*), on the logarithm of geographic distance (Table 2). Isolation by distance was also observed for ordered haplotypes, with the same taxa, except *Strombosiopsis tetrandra*, showing a negative regression slope, *blog*(N*_ij_*). A phylogeographic structure was only observed in three taxa: *Erythrophleum ivorense/suaveolens*, *Greenwayodendron suaveolens* subsp. *suaveolens* var. *suaveolens* and *Symphonia globulifera.*

**Table 2:** Tests of population expansion and patterns of isolation by distance in 14 Afrotropical tree taxa.

	*P-value,* H0:Demographicexpansion	*P-value,* H0:Spatialexpansion	*blog*(*F*_ij_)	*P*(IBD_*F*_ij_)	*blog*(*N*_ij_)	*P*(IBD_*N*_ij_)	*P*(blog(*N*_ij_)<*blog*(*N*_ij_perm.))
*Dataset 1*							
*Anthonotha macrophylla*	*	ns	−0.167	***	−0.196	***	ns
*Baillonella toxisperma*	*	*	−0.179	***	−0.176	**	ns
*Carapa parviflora*	ns	ns	−0.093	***	−0.153	***	ns
*Ceiba pentandra*	nc	nc	nc	nc	nc	nc	nc
*Coula edulis*	ns	ns	0.002	ns	0.014	ns	ns
*Diospyros mannii*	*	ns	0.007	ns	0.059	ns	ns
*Erythrophleum ivorense/suaveolens*	*	+	−0.157	***	−0.231	***	*
*Greenwayodendron suaveolens*	ns	ns	−0.238	***	−0.181	**	ns
*Milicia excelsa*	ns	*	−0.185	**	−0.169	**	ns
*Panda oleosa*	nc	nc	nc	nc	nc	nc	nc
*Scorodophloeus zenkeri*	*	ns	−0.144	***	−0.147	*	ns
*Strombosiopsis tetrandra*	ns	ns	−0.147	***	−0.037	ns	ns
*Symphonia globulifera*	+	ns	−0.103	***	−0.175	***	***
*Trichoscypha acuminata*	ns	ns	−0.073	*	−0.067	+	ns
*Dataset 2*							
*Greenwayodendron suaveolens* subsp.suaveolens var. suaveolens (Engl.& Diels) Verdc.	*	***	−0.268	***	−0.401	***	**
*Milicia excelsa* (Welw.) C.C. Berg	+	**	−0.096	***	−0.179	***	ns
*Symphonia globulifera* L.f.	*	ns	−0.126	***	−0.173	***	**
*Trichoscypha acuminata* Engl.	*	***	−0.093	***	−0.092	***	ns

*blog*(*F*_ij_) and *P*(IBD_*F*_ij_), slope and test of isolation by distance; *blog*(*N*_ij_) and *P*(IBD_*N*_ij_) slope and test of isolation by distance using ordered alleles; *P*(blog(*N*_ij_)<blog(*N*_ij_perm.)), test for phylogeographic structure. For details, see materials and methods. +, *P*<0.1; *, *P*<0.05; **, *P*<0.01; ***, *P*<0.00; ns, not significant; nc, not calculated.

### Patterns of Endemism and Differentiation

In dataset 1, there was no significant difference of genetic diversity statistics between regions in any of the two evolutionary scenarios examined; however, some diversity statistics (*h*, *Npriv* and *Ar_3_*) were found to differ significantly among taxa (Table 3). Genetic diversity, numbers of haplotypes and private haplotypes were generally similar between regions defined according to both evolutionary scenarios (Table 4, Table 5). Only in *M. excelsa* in dataset 2, genetic diversity was larger in the S than in the N regions (non-overlapping 95% confidence intervals based on mean ±1.96 * SE). In dataset 2, private rarefied allelic richness *Arpriv*_18_ was numerically larger in all four species in the E than in the W, whereas no common trend was observed in the N-S scenario. Negative regression slopes with latitude were observed for within-population allelic richness (*Ar*_3_) in *M. excelsa* and for within-population genetic diversity *h* in *S. globulifera*, i.e. southern populations of these species had higher diversity statistics (Table S3). Differentiation estimated as *F*_ST_ or *N*_ST_ between regions in dataset 1 was significant (*P*<0.05) for four or five taxa in the W-E scenario and for three or four taxa in the N-S scenario (Table 6). *Erythrophleum ivorense/suaveolens* and *Scorodophloeus zenkeri* showed W-E but not N-S differentiation, and *S. globulifera* displayed strong N-S, but only weak W-E differentiation; patterns were less clear for the remaining species. In dataset 2, all four species showed significant N-S differentiation, but only two showed W-E differentiation (Table 6). A contribution of mutations to differentiation (*N*_ST_ *>N*_ST(perm.)_) was detected in *Erythrophleum ivorense/suaveolens* and *Anthonotha macrophylla* (W-E) and in *G. suaveolens subsp. suaveolens var. suaveolens* (N-S, dataset 2).

**Table 3:** Analysis of variance in 14 Afrotropical tree taxa under two evolutionary scenarios.

	*W-E scenario*				*N-S scenario*			
	Taxon		Region		Taxon		Region	
	*F*	*Pr(>F)*	*F*	*Pr(>F)*	*F*	*Pr(>F)*	*F*	*Pr(>F)*
*Nhap*	2.134	0.086	0.131	0.72	2.300	0.068	0.544	0.467
*Npriv*	2.936	0.028*	0.105	0.749	3.215	0.019*	0.426	0.52
*Ar_3_*	2.543	0.048*	0.038	0.848	3.285	0.018*	0.009	0.926
*h*	2.662	0.040*	0.003	0.954	3.655	0.011*	0.000	0.997
*v*	0.640	0.786	0.233	0.633	1.133	0.408	0.280	0.601
*N*	0.263	0.989	0.827	0.372	0.261	0.990	3.908	0.059

*F* values and significance levels are given: *, P<0.05; **, P<0.01; ***, P<0.001. *Nhap*, number of haplotypes; *Npriv*, number of private haplotypes; *Ar_3_*, haplotype richness in a random sample of 3 sequences; genetic diversity with unordered (*h*) or ordered haplotypes (*v*); *N*, sample size.

**Table 4:** Diversity estimates across 14 Afrotropical tree taxa under two evolutionary scenarios (dataset 1).

	Population	N	Nhap	Ar_80_	Npriv	Arpriv_80_
*W-E scenario*	West	118	39	33.3	27	22.2
	East	103	36	32.9	24	22.0
*N-S scenario*	North	95	36	34.0	21	21.0
	South	126	42	33.8	27	20.8

*N*, sample size; *Nhap*, number of haplotypes; *Ar_80_*, rarefied haplotype richness in a random sample of 80 sequences; *Npriv*, number of private haplotypes; *Arpriv_80_*, rarefied private haplotype richness in a random sample of 80 sequences.

**Table 5:** Diversity estimates in 14 Afrotropical tree taxa under two evolutionary scenarios.

	W-E scenario												N-S scenario											
	West (coast)						East (inland)						North						South					
W-E environmental gradient	*N*	*h(SE)*	*Nhap*	*Ar*	*Npriv*	*Arpriv_18_*	*N*	*h(SE)*	*Nhap*	*Ar*	*Npriv*	*Arpriv_18_*	*N*	*h(SE)*	*Nhap*	*Ar_3_*	*Npriv*	*Arpriv_18_*	*N*	*h(SE)*	*Nhap*	*Ar_3_*	*Npriv*	*Arpriv_18_*
**Dataset 1**																								
*Anthonotha macrophylla*	11	0.764(0.083)	4	2.34	3		6	0.800(0.122)	3	2.40	2		8	0.714(0.123)	3	2.21	3		9	0.556(0.165)	3	1.91	3	
*Baillonella toxisperma*	3	0.667(0.314)	2	2.00	1		10	0.600(0.131)	3	1.98	2		6	0.733(0.155)	3	2.25	2		7	0.286(0.196)	2	1.43	1	
*Carapa parviflora*	10	0.511(0.164)	3	1.83	3		7	0.857(0.102)	4	2.57	4		10	0.644(0.152)	4	2.10	2		7	0.905(0.103)	5	2.71	3	
*Ceiba pentandra*	6	0.000(0.000)	1	1.00	0		5	0.000(0.000)	1	1.00	0		5	0.000(0.000)	1	1.00	0		6	0.000(0.000)	1	1.00	0	
*Coula edulis*	14	0.143(0.119)	2	1.21	1		4	0.000(0.000)	1	1.00	0		6	0.333(0.215)	2	1.50	1		12	0.000(0.000)	1	1.00	0	
*Diospyros mannii*	9	0.222(0.166)	2	1.33	1		5	0.400(0.237)	2	1.60	1		2	0.000(0.000)	1	nc	0		12	0.318(0.164)	3	1.50	2	
*Erythrophleum ivorense/suaveolens*	11	0.346(0.172)	3	1.55	3		9	0.806(0.120)	5	2.46	5		12	0.803(0.078)	5	2.45	4		8	0.643(0.184)	4	2.11	3	
*Greenwayodendron suaveolens*	8	0.714(0.123)	3	2.21	1		8	0.714(0.123)	3	2.21	1		2	0.000(0.000)	1	nc	0		14	0.747(0.078)	4	2.31	3	
*Milicia excelsa*	3	0.000(0.000)	1	1.00	0		13	0.538(0.060)	2	1.81	1		9	0.500(0.128)	2	1.75	0		7	0.286(0.196)	2	1.43	0	
*Panda oleosa*	5	0.000(0.000)	1	1.00	0		7	0.000(0.000)	1	1.00	0		4	0.000(0.000)	1	1.00	0		8	0.000(0.000)	1	1.00	0	
*Scorodophloeus zenkeri*	4	0.667(0.204)	2	2.00	2		9	0.778(0.110)	4	2.38	4		3	0.667(0.314)	2	2.00	1		10	0.867(0.071)	5	2.61	4	
*Strombosiopsis tetrandra*	11	0.891(0.063)	6	1.68	5		5	0.000(0.000)	1	1.00	0		11	0.818(0.083)	5	2.49	4		5	0.600(0.175)	2	1.90	1	
*Symphonia globulifera*	13	0.795(0.085)	6	2.43	5		6	0.933(0.122)	5	2.80	4		9	0.694(0.147)	4	2.20	4		10	0.778(0.137)	6	2.42	6	
*Trichoscypha acuminata*	10	0.600(0.131)	3	1.98	2		9	0.000(0.000)	1	1.00	0		8	0.429(0.169)	2	1.64	0		11	0.345(0.172)	3	1.55	1	
Average	8.43	0.451	2.79	1.68	1.93		7.36	0.459	2.57	1.80	1.71		6.79	0.453	2.57	1.88	1.50		9.00	0.452	3.00	1.78	1.93	
N species with larger valueWest or North	9	4	5	4	5								4	7	4	7	4							
N species with larger valueEast or South	4	7	5	7	5								10	5	6	3	6							
**Dataset 2**																								
*Greenwayodendron suaveolens* subsp. *suaveolens var. suaveolens (Engl. & Diels) Verdc.*	80	0.648(0.036)	7	4.03	3	1.70	89	0.540(0.055)	7	4.03	3	1.71	61	0.585(0.059)	5	3.97	2	2.05	108	0.598(0.026)	8	3.61	4	1.70
*Milicia excelsa* (Welw.) C.C. Berg	28	0.349(0.090)	2	2	0	0.00	99	0.415(0.042)	3	2.18	1	0.18	85	0.266(0.056)	3	2.17	1	0.21	42	0.512(0.017)	2	2.00	0	0.05
*Symphonia globulifera* L. f.	48	0.847(0.029)	14	7.98	12	6.61	18	0.909(0.044)	10	10	8	8.63	35	0.822(0.067)	8	6.48	7	6.03	31	0.805(0.042)	15	9.99	14	9.54
*Trichoscypha acuminata* Engl.	31	0.582(0.093)	3	2.83	0	0.05	42	0.582(0.076)	5	4.30	2	1.52	34	0.451(0.060)	2	2	0	0.28	39	0.529(0.084)	5	4.14	3	2.43

*N*, sample size; *h(SE)*, gene diversity and its standard error; *Nhap*, number of haplotypes; *Ar*, rarefied haplotype richness in a random sample of 3 sequences in dataset 1 or 18 sequences in dataset 2; *Npriv*, number of private haplotypes; *Arpriv*, rarefied private haplotypic richness; nc, not calculated.

**Table 6:** Differentiation patterns in 14 Afrotropical tree taxa under two evolutionary scenarios.

	W-E scenario					N-S scenario				
Taxon	F_ST_		N_ST_		P(N_ST_>N_ST(perm)_)	F_ST_		N_ST_		P(N_ST_>N_ST(perm)_)
**Dataset 1**										
*Anthonotha macrophylla*	0.113	+	0.313	**	*	0.368	**	0.336	**	
*Baillonella toxisperma*	0.223	+	0.342	*		0.719	**	0.316	**	
*Carapa parviflora*	0.333	***	0.139	**		0.149	*	0.062		
*Ceiba pentandra*	nc		nc		nc	nc		nc		nc
*Coula edulis*	−0.130		0.000		nc	0.12		0.000		nc
*Diospyros mannii*	−0.051		−0.062			−0.283		0.040		
*Erythrophleum ivorense/suaveolens*	0.439	***	0.660	***	*	0.081		0.062		
*Greenwayodendron suaveolens*	0.048		0.102			0.196		0.192		
*Milicia excelsa*	0.297		0.500			0.353	+	0.365	+	
*Panda oleosa*	nc		nc		nc	nc		nc		nc
*Scorodophloeus zenkeri*	0.265	**	0.349	**		0.115		0.232	+	
*Strombosiopsis tetrandra*	0.335	**	0.280	+		0.074		0.257		
*Symphonia globulifera*	0.098	+	0.146	+		0.263	***	0.491	***	+
*Trichoscypha acuminata*	0.233	+	0.167			−0.061		−0.025		
Number of tests with P<0.05	4		5		2	4		3		0
Number of tests with P<0.10	8		7		3	5		6		1
**Dataset 2**										
*Greenwayodendron suaveolens* subsp. *suaveolens var*. *suaveolens* (*Engl*. *& Diels*) *Verdc*.	0.180	***	0.154	***		0.186	***	0.299	***	*
*Milicia excelsa* (Welw.) C.C. Berg	−0.013		−0.009			0.255	***	0.220	***	
*Symphonia globulifera* L. f.	0.111	***	0.148	**		0.185	***	0.252	***	
*Trichoscypha acuminata* Engl.	−0.005		−0.013			0.079	*	0.156	***	

Estimates of differentiation statistics are followed by results from one-sided permutation tests for population structure. +, *P*<0.10; *, *P*<0.05; **, *P*<0.01; ***, *P*<0.001; ns, not significant; nc, not calculated.

Simulations showed that a population divergence scenario with mean levels of diversity and differentiation similar to those in dataset 1 and sample sizes of n = 8 per deme, i.e. simulated scenario A3, would lead to 26% significant differentiation tests at a level of α = 0.05 based on *F*_ST_ (Table 7, Table S4). In dataset 1, 29% (8 out of 28) differentiation tests were significant. If we assume that the variation associated to sampling different taxa is analogous to the stochastic variation in the coalescent process, this would suggest that dataset 1 had a detection power of population genetic structure similar to the one expected in a realistic differentiation scenario. In a differentiation scenario with larger sample sizes, n = 30 per deme (scenarios A8 or A9), the detection power of genetic differentiation rose to 73%, indicating that differentiation tests in dataset 2 were about three times more likely to detect a differentiation signal than tests in dataset 1. In the modified A3 scenario (sampling four samples in each of four demes), inter-population differentiation was larger than differentiation between groups containing demes from distinct populations, *F*_CT[(01)(23)]_>*F*_CT[(02)(13)]_, in 58% of the cases and *F*_CT[(01)(23)]_ ≥ *F*_CT[(02)(13)]_ in 98% of the cases. This suggested that the power of dataset 1 to distinguish differentiation patterns produced by alternative evolutionary scenarios was at least 58% (in 40% of cases no difference in differentiation patterns was detected, and in only 2% of cases a reversed differentiation pattern appeared). In simulations without genetic structure (B1–B4), only 0–4% of the tests detected a differentiation signal. This suggested a low level of false positive differentiation tests even for the small sample sizes of dataset 1.

**Table 7:** Simulation results showing summary statistics and the expected proportion of significant differentiation tests under divergence or constant population size scenarios for simulated deme sizes, *N*, and mutation rates, *µ*, and their comparison to observed data from dataset 1.

	Fastsimcoal parameters					Summary statistics					
	*N*	*t1*	*t2*	*t3*	*µ*	*n/pop*	*haps/pop*	*S*	*h*	*F*_ST_	Fraction of sampleswith significant *F*_ST_ a
Dataset 1						7.89	2.73	5.80	0.490	0.166	0.29
Divergence A1	10000	1	200	1600	2.00E-08	8	2.55	2.93	0.465	0.052	0.08
Divergence A2	10000	1	200	1600	5.00E-08	8	3.68	6.90	0.661	0.055	0.10
Divergence A3	2000	1	200	1600	1.00E-07	8	2.45	3.38	0.442	0.187	0.26
Divergence A4	1000	1	200	1600	5.00E-08	8	1.85	1.74	0.305	0.215	0.17
Divergence A5	1000	1	200	1600	1.00E-07	8	2.05	2.45	0.339	0.253	0.38
Divergence A6	1000	1	200	1600	5.00E-07	8	3.62	10.17	0.649	0.430	0.85
Divergence A7	5000	1	200	1600	1.00E-07	8	3.72	8.34	0.687	0.115	0.23
Divergence A8	5000	1	200	1600	1.00E-07	30	6.17	11.86	0.686	0.129	0.73
Divergence A9	2000	1	200	1600	1.00E-07	30	3.53	5.59	0.439	0.190	0.73
Constant B1	1000	1			1.00E-07	8	1.98	1.57	0.352	−0.007	0.00
Constant B2	2000	1			1.00E-07	8	2.23	1.96	0.417	0.002	0.01
Constant B3	2000	1			1.00E-06	8	4.73	12.64	0.794	−0.007	0.04
Constant B4	2000	1			1.00E-06	30	9.12	17.13	0.798	−0.006	0.04

Averages over 100 independent simulations are shown, see Figure 4 and Table S4.

^a^ The fraction of samples with significant *F*_ST_ considers the proportion of 1-sided tests with *P*<0.05.

### Shared Spatial Patterns of Genetic Structure

**Figure 5:**
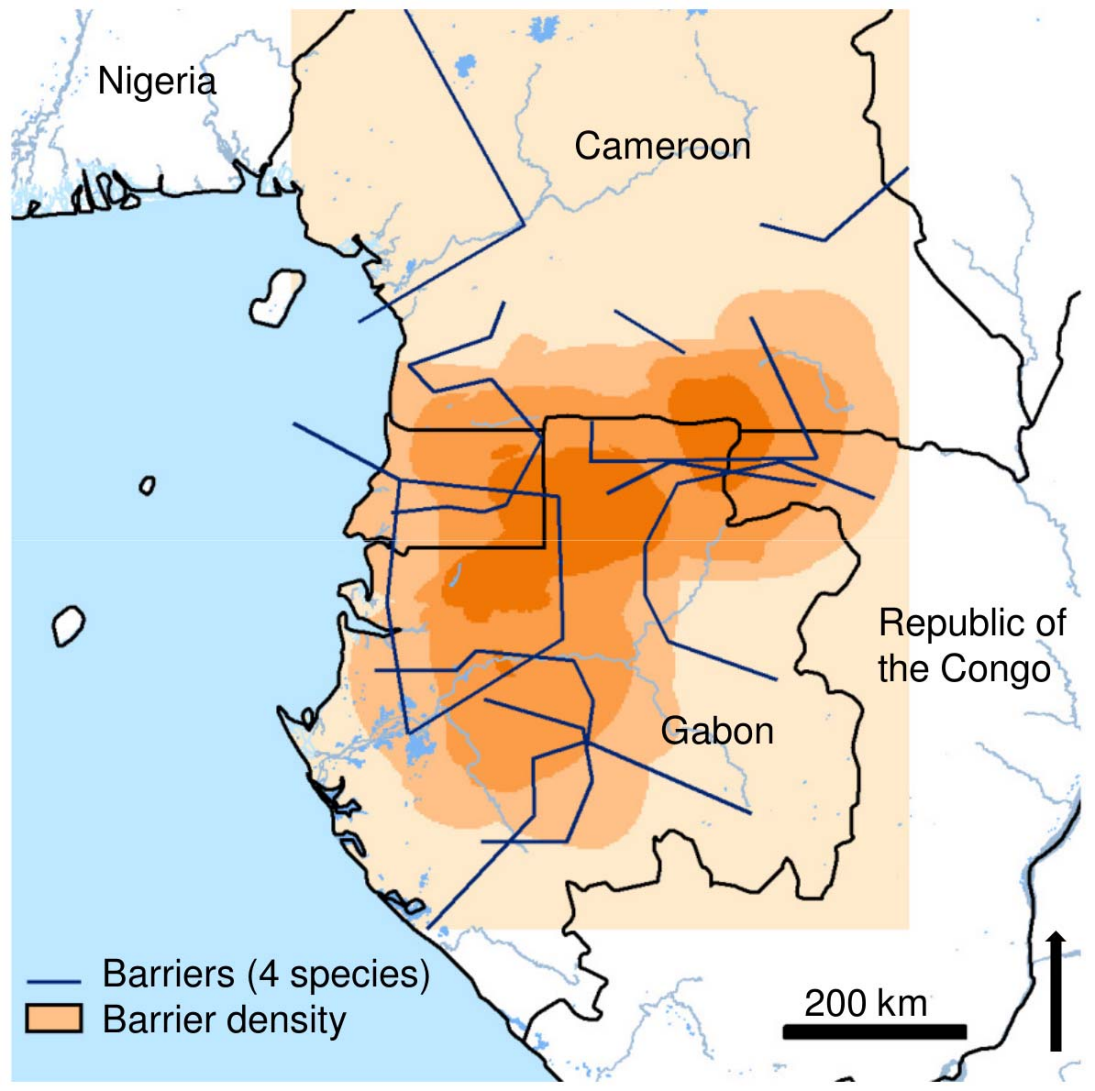
Graphical representation of co-location of the three strongest genetic barriers (barrier density) observed across four Afrotropical tree species (dataset 2). Barriers (in blue) are based on the pair-wise differentiation (*G*_ST_) among sampling locations. More intense coloration indicates higher barrier density.

The barrier density analysis based on the four species of dataset 2 identified a region of barrier co-location separating the N and the S of the LG study region between ca. 0 and 3° N, at the latitude of Equatorial Guinea, coinciding to some extent with the differentiation expected under the N-S scenario (Figure 5).

## Discussion

This study shows that fourteen common Afrotropical rainforest tree taxa (dataset 1) exhibited variation in their patterns of genetic endemism and differentiation and did not show clear majority support for any of two simple evolutionary scenarios in response to physio-climatic features in the Lower Guinea, i.e., 1) the W-E environmental gradient or 2) the N-S seasonal inversion. This observation allows several possible interpretations: 1) absence of a common pattern, or non-detection of a common pattern because it was too weak to be detected with the available sample sizes; 2) existence of a common genetic structure pattern but only for a subset of the study taxa; or 3) a confounding effect due to sampling ill-defined taxonomic species. We applied coalescent simulations and complementary sampling in a sub-sample of four of the fourteen taxa in order to further examine these possible interpretations and to clarify our results. Interestingly, the complementary sampling distinguished an N-S differentiation pattern shared among the four species with larger samples.

One caveat of our study is that the tree flora of Central African rainforests is not sufficiently well known to allow unambiguous species identification. The taxa considered in dataset 1 are species-level taxa according to current taxonomic classifications (except *Erythrophleum ivorense/suaveolens*) but some are most probably complexes of species that cannot be distinguished easily in the field (see material and methods) and, in any case, it cannot be taken for granted that any of the taxa considered in dataset 1 matches the biological species concept. Our study approach should generally remain valid for species complexes because we make no assumption on whether evolutionary forces affecting diversification led to differentiated populations or to distinct species. Indeed, the genealogy of a non-recombinant DNA fragment, as studied here, is not directly dependent on speciation processes [47], [76]. In species complexes, confounding effects on the phylogeographic pattern could arise from sampling bias associated to small sample sizes, or alternatively, from specific geographically circumscribed evolutionary processes, such as introgression in one of the range parts. It is however also possible that a morphological taxon harbours genetically well-differentiated biological species, and their inadvertent lumping in a single data set can cause misinterpretation of the phylogeographic pattern: because woody plant lineages experience generally slower diversification and speciation rates than herbaceous plants [77], sister species lumped together may actually have diverged before the Plio-Pleistocene. In this case, genetic footprints of ancient evolutionary history could erroneously be interpreted as resulting from recent (Plio-Pleistocene) demographic or adaptive processes.

Such confounding effects related to species discrimination problems are perhaps more likely for taxa with deep lineage divergence among haplotypes. We observed deep lineage divergence (see haplotype networks in Fig. 1) in *Strombosiopsis tetrandra* and *Anthonotha macrophylla* which may potentially harbour distinct yet undescribed biological species (F.J. Breteler, pers. com.) suggesting that further research is needed on species delimitation within these taxa, and also in *Erythrophleum ivorense/suaveolens*. *Erythrophleum ivorense* and *E. suaveolens* are sister species which cannot easily be distinguished based on vegetative characters and whose geographic ranges (coastal *vs.* inland) have only recently been inferred based on a phylogeographic study that compared the distribution of genetic diversity of vegetative specimens with the reproductive morphology in a few fertile specimens [34]. When we excluded the cited taxa to control for the possible confounding effects, we still did not find majority support for any of the two simple evolutionary scenarios (results not shown).

Our data did not find support for a common pattern of allelic endemism across species in dataset 1, as the numbers of private alleles were low and similar between regions in both scenarios. In dataset 2, there was a weak tendency for eastern locations to contain (numerically) more endemic alleles than western locations. This suggested that there was no general pattern in either region to harbour consistently older populations. If the W-E environmental gradient had enabled persistence of rainforest taxa in the moister Atlantic zone only during past climate oscillations [18], [33], higher allele endemism would have been expected in the W only. Our results did not concur with this expectation.

Our simulation approach indicated that the power to detect a common differentiation pattern across taxa (with significant *F*_ST_ tests) was *ca*. 26% in dataset 1 and *ca*. 73% in dataset 2, given the level of differentiation observed in our data. In dataset 1, three to five out of 14 taxa showed significant population differentiation compatible with the proposed W-E or N-S scenarios, which was close to the expected proportion from simulations. The comparison of *F*_CT_ values between the two alternative groupings of simulated demes indicated that *F*_CT_ was (strictly) larger in the true differentiation scenario in 58% of the cases vs. only 2% in the false scenario in samples analogous to those of dataset 1. This approach, i.e., the comparison of *F*_ST_ values between alternative groupings of our samples was therefore a more powerful approach to detect the correct scenario than *F*_ST_ tests. Our data displayed, respectively, six *F*_ST_ values larger in the W-E than in the N-S scenario, and six values larger in the reverse comparison. This suggested that, given the power of dataset 1, there was no majority support for neither of the two hypothesized scenarios. Simulations also showed that the strength of the differentiation signal had a strong effect on the power (Table 7): in the simulated scenario (A6) with mean *F*_ST_ = 0.43, sample sizes of n = 8 per deme had 85% power to detect differentiation. We can therefore conclude that our taxa from dataset 1 probably had idiosyncratic histories, or, if they had been exposed to the same evolutionary scenario, the differentiation effect was not strong enough, and/or there was too high population substructure, to make it detectable as a common pattern across taxa. In dataset 2, all four species exhibited N-S differentiation, but only two showed W-E differentiation. This suggests that a common N-S pattern might exist, at least across the four examined species, and this result was also supported by the barrier density analysis (see below). Simulations also indicated that we could confidently discard the hypothesis that all taxa would exhibit panmixia in the LG since the rate of false positive differentiation tests was low in simulations (0–4%).

The apparent idiosyncratic genetic structure patterns observed in dataset 1 could partially be explained by the lack of a common pattern of bottlenecks or postglacial expansion, which contrasts with comparative phylogeographic studies in other regions of the world [11], [78]. The only common pattern across taxa that we could observe with some degree of confidence in dataset 1 was isolation by distance within taxa, which is expected when dispersal is limited in space [79]. Dataset 2 showed that IBD patterns were detected similarly in the corresponding taxa of both datasets, and the visual comparison of haplotype maps also suggested that the small datasets provided fair information on haplotype diversity and its geographic organization.

The power to detect differentiation patterns was considerably improved in dataset 2 (see above) and a common genetic break zone was identified by the barrier density analysis separating the N from the S of the LG for these taxa (*G. suaveolens* subsp. *suaveolens* var. *suaveolens*, *M. excelsa*, *S. globulifera* and *T. acuminata*), in agreement with the expectation of the N-S evolutionary scenario. A similar genetic break zone in the LG region has been suggested based on nuclear markers in a review by Hardy et al. [36] which included also the following species: *Santiria trimera* [45], *Distemonanthus benthamianus* [44], *Irvingia gabonensis* [43] and *Aucoumea klaineana* [42], and moreover, floristic analysis on local assemblages of tree species also suggested the existence of a floristic differentiation between N and S of the climatic hinge [37]. Our suggested barrier zone was located somewhat further south than that suggested by Hardy et al. [36]. That paper offers several non-excluding explanations for a divergence pattern matching the climatic hinge, namely 1) a modified glacial refuge hypothesis where Plio-Pleistocene populations of forest organisms persisted through adverse climate stages on both sides of the climatic hinge, 2) impeded gene flow between regions caused by phenological differences, potentially coupled with 3) postzygotic selection affecting ill-adapted immigrants into either regions, which could occur if northern and southern populations were locally adapted to their idiosyncratic climatic conditions [17]. These hypotheses await further testing.

More generally, it remains to be examined which is the importance of the climatic hinge or other physio-climatic features in structuring the genetic diversity of the LG flora. Our study, with the lack of a common pattern using small multi-taxa datasets, and congruent or partially congruent differentiation patterns according to two scenarios in larger datasets of fewer species, suggests that in-depth comparative phylogeographic work across a wide range of taxa requires a good understanding of evolutionary processes between closely related species and in species complexes. More detailed population genetic case studies are therefore necessary, including studies on species complexes, before specific physio-climatic or species-inherent features (e.g., climatic tolerance, mating system or substrate affinity [13], [80]–[82]) can be proposed as drivers of any shared phylogeographic patterns in African tropical rainforest trees.

## Supporting Information

Table S1 **Geographical location, sample and voucher information of the plant material included in dataset 1.**
(XLSX)

Table S2 **Life history traits of 14 African rainforest tree taxa.**
(DOCX)

Table S3 **Diversity gradients at the** ***trnC-ycf6*** **region in four Afrotropical tree species.**
(DOCX)

Table S4 **Details on coalescent simulation results.**
(XLSX)
