# Supplementary material for: Comparative Phylogeography in Rainforest Trees from Lower Guinea, Africa
Source: PLoS One. 2014 Jan 8;9(1):e84307. doi: 10.1371/journal.pone.0084307 (PMC3885573; doi:10.1371/journal.pone.0084307)
Supplement: Table S2 — Life history traits of 14 African rainforest tree taxa. (DOCX) [file pone.0084307.s002.docx]

Table S2. Life history traits of 14 African rainforest tree taxa.

| Taxon | Dominance | Distri-bution | Seed dispersal | Succession |
| --- | --- | --- | --- | --- |
| *Anthonotha macrophylla* | dominated | GC | P, G | early - intermediate |
| *Baillonella toxisperma* | emergent | UG+LG | Ro, E, Md | late |
| *Ceiba pentandra* | emergent | AA | W | early |
| *Carapa parviflora* | codominant | AA | Ro | late |
| *Coula edulis* | codominant | UG+LG | P, E? | late |
| *Diospyros mannii* | dominated - codominant | UG+LG | E, Me | late |
| *Erythrophleum ivorense/suaveolens* | emergent | GCp | P, Md | early |
| *Greenwayodendron suaveolens* | codominant | LG+C | B, M, E | late |
| *Milicia excelsa* | emergent | GCp | Bat, B | early |
| *Panda oleosa* | codominant | GC | P, E?, Ro, Ru | early |
| *Scorodophloeus zenkeri* | codominant | LG+C | P, G | late |
| *Strombosiopsis tetrandra* | codominant | LG+C | Ro, Ru, E | late |
| *Symphonia globulifera* | codominant | AA | B, M | late |
| *Trichoscypha acuminata* | dominated - codominant | LG+C | M | late |

Distribution: GC, Guineo-Congolian; GCp, Guineo-Congolian region and its periphery; LG, Lower Guinea; AA, America and Africa; UG+LG, Upper and Lower Guinea; LG+C, Lower Guinea and Congolia. Seed dispersal: P, predated; G, gravity; Ro, rodent; E, elephant; M, monkey; Md, dispersal by monkeys through handling seeds; Me, dispersal by monkeys through endozoochory; W, wind; B, bird; Ru, ruminant; RRE, rodent, ruminant, elephant syndrome; BM, bird-monkey syndrome.
