# Supplementary material for: Comparative Phylogeography in Rainforest Trees from Lower Guinea, Africa
Source: PLoS One. 2014 Jan 8;9(1):e84307. doi: 10.1371/journal.pone.0084307 (PMC3885573; doi:10.1371/journal.pone.0084307)
Supplement: Table S3 — Diversity gradients at the trnC-ycf6 region in four Afrotropical tree species. (DOCX) [file pone.0084307.s003.docx]

Table S3. Diversity gradients at the *trnC-ycf6* region in four Afrotropical tree species. Allelic richness Ar3 and genetic diversity h at the level of sampling site were regressed on latitude or longitude. Regression slopes, b, and the coefficient of determination, R2, are reported. Values with R2 >0.2 are indicated in bold. *, *P*<0.05; all other tests were not significant.

|  | Ar3 - Latitude | | h - Latitude | | Ar3 - Longitude | | h - Longitude | |
| --- | --- | --- | --- | --- | --- | --- | --- | --- |
|  | b | R2 | b | R2 | b | R2 | b | R2 |
| *Greenwayodendron suaveolens* subsp. suaveolens var. suaveolens (Engl. & Diels) Verdc. | -0.052 | 0.022 | -0.027 | 0.040 | -0.035 | 0.006 | -0.024 | 0.020 |
| *Milicia excelsa* (Welw.) C.C. Berg | **-0.122** | **0.470*** | 0.016 | 0.020 | 0.013 | 0.006 | -0.013 | 0.010 |
| *Symphonia globulifera* L.f. | **-0.113** | **0.272** | **-0.108** | **0.453*** | **0.094** | **0.211** | 0.047 | 0.073 |
| *Trichoscypha acuminata* Engl. | -0.022 | 0.011 | 0.011 | 0.003 | 0.032 | 0.010 | 0.042 | 0.023 |
